# Beached bachelors: An extensive study on the largest recorded sperm whale *Physeter macrocephalus* mortality event in the North Sea

**DOI:** 10.1371/journal.pone.0201221

**Published:** 2018-08-07

**Authors:** Lonneke L. IJsseldijk, Abbo van Neer, Rob Deaville, Lineke Begeman, Marco van de Bildt, Judith M. A. van den Brand, Andrew Brownlow, Richard Czeck, Willy Dabin, Mariel ten Doeschate, Vanessa Herder, Helena Herr, Jooske IJzer, Thierry Jauniaux, Lasse Fast Jensen, Paul D. Jepson, Wendy Karen Jo, Jan Lakemeyer, Kristina Lehnert, Mardik F. Leopold, Albert Osterhaus, Matthew W. Perkins, Uwe Piatkowski, Ellen Prenger-Berninghoff, Ralf Pund, Peter Wohlsein, Andrea Gröne, Ursula Siebert

**Affiliations:** 1 Faculty of Veterinary Medicine, Department of Pathobiology, Utrecht University, Utrecht, The Netherlands; 2 Institute for Terrestrial and Aquatic Wildlife Research, University of Veterinary Medicine Hannover, Foundation, Büsum, Germany; 3 Institute of Zoology, Zoological Society of London, Regent’s Park, London, United Kingdom; 4 Department of Viroscience, Erasmus University Medical Centre, Rotterdam, The Netherlands; 5 Scottish Marine Animal Stranding Scheme, SRUC Veterinary Services, Inverness, Scotland, United Kingdom; 6 Wadden Sea National Park Authority of Lower Saxony, Wilhelmshaven, Germany; 7 Observatoire PELAGIS, University of La Rochelle - CNRS, La Rochelle, France; 8 Department of Pathology, University of Veterinary Medicine Hannover, Hannover, Germany; 9 Faculty of Veterinary Medicine, Department of Morphology and Pathology, University of Liège, Liège, Belgium; 10 Aalborg University, Department of Chemistry and Bioscience - Section for Environmental Technology, Aalborg, Denmark; 11 Research Center for Emerging Infections and Zoonoses (RIZ), University of Veterinary Medicine Hannover, Hannover, Germany; 12 Wageningen Marine Research, Den Helder, The Netherlands; 13 GEOMAR, Helmholtz Centre for Ocean Research Kiel, Kiel, Germany; 14 Institut für Hygiene und Infektionskrankheiten der Tiere, Justus-Liebig-Universität Giessen, Gießen, Germany; 15 Lower Saxonian State Office for Consumer Protection and Food Safety (LAVES), Cuxhaven, Germany; Hawaii Pacific University, UNITED STATES

## Abstract

Between the 8^th^ January and the 25^th^ February 2016, the largest sperm whale *Physeter macrocephalus* mortality event ever recorded in the North Sea occurred with 30 sperm whales stranding in five countries within six weeks. All sperm whales were immature males. Groups were stratified by size, with the smaller animals stranding in the Netherlands, and the largest in England. The majority (n = 27) of the stranded animals were necropsied and/or sampled, allowing for an international and comprehensive investigation into this mortality event. The animals were in fair to good nutritional condition and, aside from the pathologies caused by stranding, did not exhibit significant evidence of disease or trauma. Infectious agents were found, including various parasite species, several bacterial and fungal pathogens and a novel alphaherpesvirus. In nine of the sperm whales a variety of marine litter was found. However, none of these findings were considered to have been the primary cause of the stranding event. Potential anthropogenic and environmental factors that may have caused the sperm whales to enter the North Sea were assessed. Once sperm whales enter the North Sea and head south, the water becomes progressively shallower (<40 m), making this region a global hotspot for sperm whale strandings. We conclude that the reasons for sperm whales to enter the southern North Sea are the result of complex interactions of extrinsic environmental factors. As such, these large mortality events seldom have a single ultimate cause and it is only through multidisciplinary, collaborative approaches that potentially multifactorial large-scale stranding events can be effectively investigated.

## Introduction

Cetacean strandings occur across all the world’s oceans and have been recorded throughout history [1]. Some stranding events attract curious crowds, and questions are often raised about potential causes. Stranding events can consist of an individual live or dead cetacean and less frequently of multiple live and/or dead animals [2]. One of the species known to be involved in such mass stranding events is the sperm whale *Physeter macrocephalus*.

The sperm whale is a widely distributed species, inhabiting deeper waters from the equator to the polar regions. Commercial whaling from the 1800’s to the 1960’s significantly reduced populations, with a global pre-whaling population estimate of 1,100,000 reduced by approximately 67% [3]. Since the moratorium on commercial whaling in the 1980’s, the current global population size is estimated at around 360,000 individuals (coefficient of variation = 0.36) with no evidence of a notable increase or reduction [3,4]. Sperm whales show distinct spatial population segregation, with females and calves normally resident year-round near breeding areas around the equator, and males migrating between the breeding areas and high-latitude feeding grounds [4,5,6,7].

Male sperm whales usually migrate along the Faeroe—Shetland Channel on their southward migration [8,9]. Some animals may enter the Norwegian trench during their southern migration and mistakenly enter the North Sea region. This is a potentially hazardous region for a pelagic species: the North Sea becomes much shallower and narrower towards its southern margin, with gradually sloping coastlines, sandbanks and tidal mudflats with high tidal amplitudes [10] (S1 Fig). Numerous historical sperm whale strandings around the North Sea have been documented, with this region being recognized as one of the global hotspots for sperm whale strandings. Smeenk [5] documented all recorded sperm whale strandings in the North Sea from 1560 until 1995. Most stranding events involved one to three sperm whales in the same location, while large mortalities in this area have been relatively uncommon [5,11].

A systematic pathological examination of single and mass stranded marine mammals may help to elucidate the cause of strandings and to investigate the general health status of marine mammal species (e.g. [12]). Standardised necropsies of cetaceans have demonstrated a wide range of infectious diseases, physical trauma, and metabolic or dietary derangements [13–15]. In addition, several direct and indirect effects of anthropogenic activities on the health of cetaceans have previously been described [16–20]. However, knowledge on the health status of sperm whales is still scarce and little pathological data has been published. Previous strandings of sperm whales have been linked to navigational errors [21]; solar storms [22,23]; climate events [24]; gastric impaction from plastic ingestion [25]; potential seismic surveys [26], contaminants [27] and disease [9,28, 29].

In early 2016, the largest sperm whale mortality event ever recorded in the North Sea region occurred, with 30 whales stranded in five countries over a period of six weeks. This stranding event allowed the systematic collection of information on the biology and health status of a large number of animals. Through a comprehensive pathological investigation, comprising gross post mortem assessment and a range of ancillary diagnostic tests, we investigated the hypothesis that this stranding event could be explained by a compromise in the health status of one or more of the stranded animals. Elimination of disease as a plausible cause of these strandings would suggest that other natural or anthropogenic factors were influential in this mortality event and we discuss potential contributing factors. In addition, and with the aim of placing this mortality event into historical context, we provide an update of the historical time series (published by Smeenk [5]) with sperm whale stranding reports from the last twenty-one years (1996–2016) in the southern North Sea region.

## Materials and methods

The animals described in this study were not used for scientific or commercial testing. All were free-living whales which died of natural causes. No consent from an Animal Use Committee is therefore required. Consequently, animal ethics committee approval was not applicable to this work.

Comprehensive gross necropsies were carried out on 23 sperm whales and additional sampling was conducted in four others. Three sperm whales could not be studied due to logistical constraints. Necropsies and histopathological studies were conducted following standard procedures [13,14,30,31].

The nutritional status was visually assessed based on the dorsal musculature and through quantitative assessment of blubber thickness, measured, where possible, immediately anterior to the dorsal fin at three locations (dorsal, lateral and ventral). Necropsies and sampling of the carcasses were performed with intervals between stranding and investigation ranging from 11 hours to eight days. Due to the significant logistical challenges in undertaking multiple beach necropsies and the rapid decomposition rate for this large species, in combination with likely peri mortem hyperthermia from live stranding, decomposition of the carcasses constrained the evaluation of the major organs and extensive histopathological assessment in most cases. The decomposition condition codes (DCC) of the carcasses at the point of necropsy were assessed following internationally standardised guidelines ([30], with DCC1 representing very fresh carcasses and DCC5 the skeletal remains of animals). The freshest animals were necropsied in the Netherlands (DCC1-3), while in Germany most carcasses were DCC4 at the time of examination. The majority of the necropsied whales in England and the one in France were DCC3-4.

Samples for histopathology were collected from all animals which were in a fresh to moderate state of decomposition (DCC1-3). Tissues available for histologic review varied from animal to animal, but included: eye (with optic nerve), skin (with any lesions), muscle, pre scapular lymph nodes, lung and associated lymph nodes, heart, liver, adrenals, kidney, oesophagus, stomachs, spleen, pancreas, intestine and associated lymph nodes, reproductive tissue and associated lymph nodes, and, if possible, brain and spinal cord. Tissues were routinely embedded in paraffin, sectioned at 4–7 mm, stained with hematoxylin and eosin (HE), and examined microscopically at the facility of origin. Additional staining was performed when appropriate, including the Periodic acid–Schiff stain to detect polysaccharides, the Von Kossa stain to quantify mineralization, and the Iron stain.

### Age determination

In 74% (20 / 27) of the cases, a mandibular tooth was collected during field sampling and subsequently analysed to determine age according to methods described [32]. In short: teeth were sectioned along the bucco-lingual plane and one half-section polished and etched in 15% formic acid until the growth layer groups could be read.

### Parasitology

Parasite presence and magnitude of infection was assessed macroscopically and rated as none (no parasites observed), mild infections, moderate infections or severe infections (following [13,33]). Parasites were collected and stored in 70% alcohol before morphological identification to genera level [34,35] using a stereomicroscope (Olympus SZ61), with additional species identification confirmed by molecular techniques in some cases. Parasitic infections were confirmed by histology and the severity of associated lesions caused by the parasites were recorded. Molecular identification was conducted after DNA isolation, PCR amplification and sequencing. Results were analysed with BioEdit (version 7.2.5) [36], MEGA 4 [37] and BLAST on GenBank [38].

### Microbiology

Samples from lung, liver, kidney, spleen, intestine, intestinal lymph nodes and any additional lesions identified at gross examination were subjected to microbiological examination, conducted according to standard protocols [39] from 15 sperm whales. Selective media was used to identify *Brucella* spp. [40].

### Virology

Virological examinations were conducted on 10% organ homogenates, made by homogenisation in transport medium (Kinematica Polytron). Total nucleic acids were isolated from 200 μl of homogenates or swab transport medium using the MagnaPure LC Total Nucleic Acid Isolation Kit (Roche Diagnostics). TaqMan RT-PCR was performed using primers and probe specific for the Influenza Matrix gene [41]. For morbillivirus reverse transcriptase PCR primers were used recognizing a phosphoprotein gene fragment [42]. For herpesvirus PCR degenerate primers, recognizing a polymerase gene fragment, were used [43]. Virus isolation was performed on the following cell lines and primary cell cultures: Vero DogSLAM [44], MDBK, TTKi, PPki, SeKC, CrFK. 100μl of 10% organ homogenates were inoculated on the different cell cultures and checked for cytopathic effects regularly, with 3 passages (7–10 days per passage) and cells and supernatants of the last passage were checked for morbillivirus and herpesvirus by PCR. Additionally, blowhole swabs from three whales were tested for herpesvirus as described above. The alignment of the partial polymerase gene (175 bp) of selected gamma- and alphaherpesviruses related mainly to cetaceans was performed with MAFFT alignment version 7 [45].

### Assessment of tympanoperiotic complexes

Twelve cases were investigated for evidence of trauma to the auditory system by investigation of the tympanic-periotic bone complexes for fractures using high-resolution computerized tomography imaging according to [46]. None of the inner ears could be investigated histologically for the presence of acoustic trauma due to logistical constraints and the fast decomposition of this tissue [47].

### Diet studies and marine litter

Stomach contents were collected and analysed according to [48]. Marine litter was investigated according to [49]. In short: gastro-intestinal tracts were opened from stomach to anus and content was collected. The gastro-intestinal tracts of seven sperm whales were also rinsed and contents sieved over 500 and 1000 μm mesh. Contents of five of these animals were in addition machine-washed (following [50]) to dissolve organic materials and isolate hard prey remains (bones, otoliths and beaks) and foreign objects (such as plastic particles). All prey items were cleaned, identified to species where possible and measured. Based on squid beak and otolith measurements, prey remains were converted into biomass, according to [51,52].

### Environmental data

A search for earthquakes with a magnitude of 4 or higher in the North Atlantic Ocean and North Sea was conducted using the online database from the European-Mediterranean Seismological Centre in the month prior to the first reported sperm whale stranding (01-12-2015–07-01-2016) (https://www.emsc-csem.org/Earthquake/?filter=yes) [53].

Data on sea surface temperature (SST) with a spatial resolution of 0.25 and respective anomalies were downloaded on 12-01-2018 from ftp://eclipse.ncdc.noaa.gov/pub/OI-daily-v2/NetCDF/ for the area ranging from N57.0-E10.0 to N68.0-E7.0. For details on the methods generating the SST data see [54]. As it is not clear when the whales entered the North Sea, daily SST values were averaged over the entire area using R Version 3.4.0 x64 [55] with the packages *raster* [56], *sp* [57] and *ncdf4* [58] and are shown with a 95% confidence interval.

### Genetics and contamination profiles

Maternal relatedness and the putative origin was investigated by studying genetic diversity of 27 of the stranded sperm whales (details in [59]). Among 24 of these individuals, contamination profiles as an indication of social structures were also investigated (details in [60]).

### Historic data

To contextualise this mortality event, we added two decades of stranding records to the data published in 1997 [5]. Stranding records were gained through national databases of Denmark (database of the University of Aarhus), the Netherlands (online database of Naturalis, Leiden; www.walvisstrandingen.nl), the United Kingdom (database of Cetacean Stranding Investigation Programme, London), Germany (database of Institute for Terrestrial and Aquatic Wildlife Research, Büsum), Belgium (database at the Belgian Marine Data Centre, Brussels) and through existing literature.

## Results

### Stranding timeline

**Fig 1:**
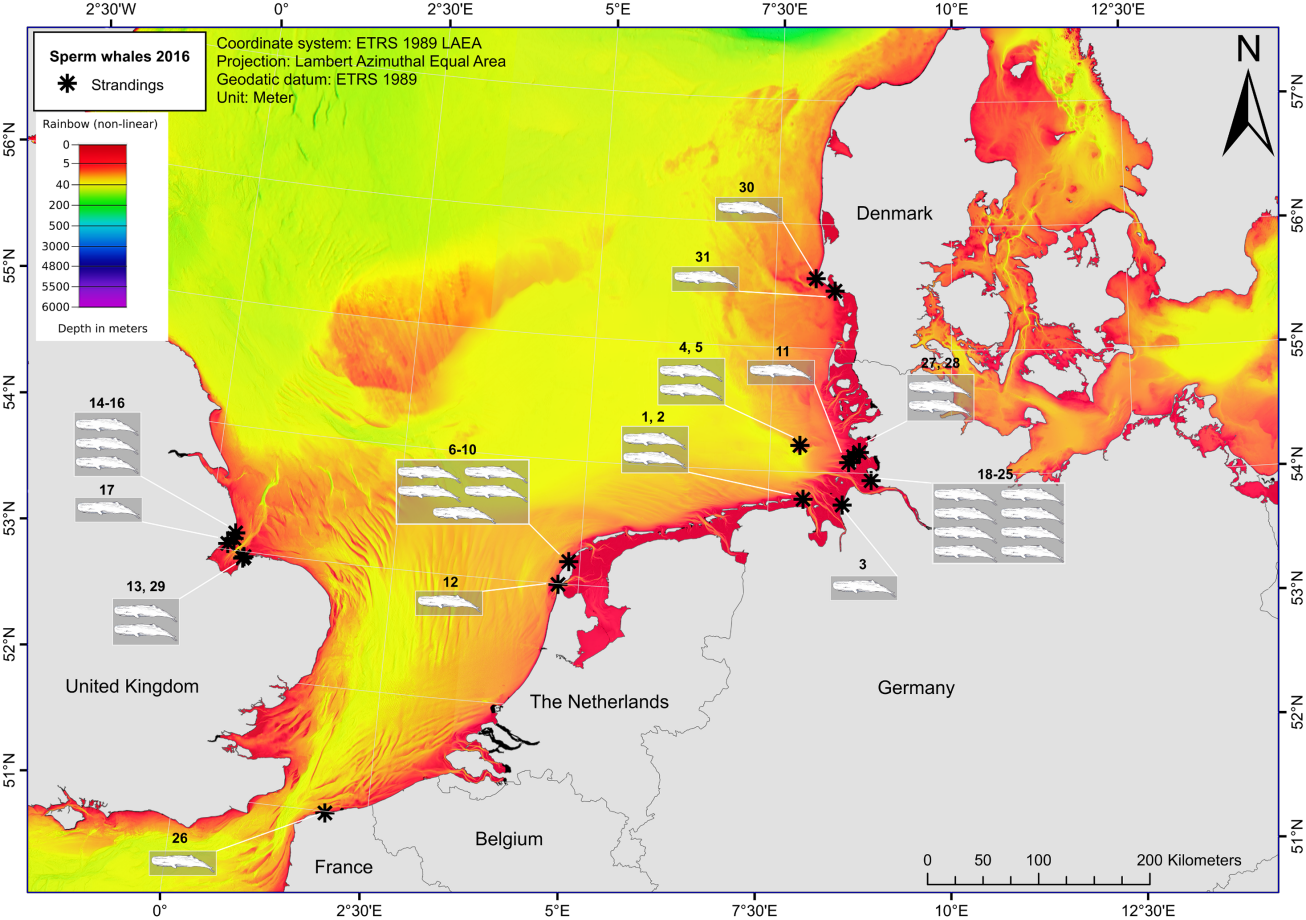
Numbers and locations of stranded sperm whales across the southern North Sea region (January-May 2016). The asterisks indicate stranding locations, with the number of the stranded sperm whales referring to the strandings data presented in S1 Table. The colour palette represents the total depth of the area, with all dark red areas being <5 m (Bathymetry layer: [61]).

The first stranded animals were found on January 8^th^ 2016 and 28 additional animals were subsequently reported up to February 21^st^ 2016: sixteen in Germany; six in England; six in the Netherlands; one in France; and one in Denmark (Fig 1; Table 1). Twenty-seven percent (8 /30) of the animals were initially found alive, but died soon after stranding.

**Table 1:** Main findings stranded sperm whales. The table included case numbers, country (DE = Germany, DK = Denmark, FR = France, NL = the Netherlands, UK = United Kingdom, here England), stranding dates (all during 2016), stranding locations, decomposition condition codes (DCC) of the cases at the time of investigation, the date of the post mortem investigation, total length (TL, in m), age (in years), any significant pre-existing disease, evidence of trauma, results of gross ear examination, visual assessment of evidence of gas emboli, detection of *Brucella* spp. and Morbillivirus infections. NE = Not Examined. NAD = No Abnormalities Detected. U = Unable to examine due to decomposition condition.

No.	Country	Date of stranding (in 2016) day-month	Lat	Lon	Live stranded observed	DCC (1–5)	Date of Postmortem day-month-year	TL (m)	Age (y)	Evidence of trauma e.g. bycatch or ship strike	Prey remains in stomach incl. cephalapod beaks	Significant pre-existing disease gross exam	Ears gross exam	Gas emboli gross exam
1	DE	8–1	53,780578	7,975660	No	5	16-1-2016	11.8	N/E	Negative	Yes	Negative	NAD	U
2	DE	8–1	53,780578	7,975660	No	4	16-1-2016	13.10	11	Negative	Yes	Negative	NAD	U
3	DE	12–1	53,741194	8,511139	No	NE	NE	NE	13	NE	NE	NE	NE	NE
4	DE	12–1	54,214610	7,913118	No	4	14-1-2016	12.0	13	Negative	Yes	Negative	NAD	U
5	DE	12–1	54,214610	7,913118	No	4	14-1-2016	12.3	13	Negative	Yes	Negative	NE	U
6	NL	12–1	53,184111	4,847167	Yes	2	13-1-2016	9.6	10	Negative	Yes	Negative	NE	NAD
7	NL	12–1	53,184111	4,847167	Yes	3	14-1-2016	11.1	16	Negative	Yes	Negative	NE	NAD
8	NL	12–1	53,184111	4,847167	Yes	3	14-1-2016	10.1	12	Negative	Yes	Negative	NE	NAD
9	NL	12–1	53,184111	4,847167	Yes	1	13-1-2016	10.25	10	Negative	Yes	Negative	NE	NAD
10	NL	12–1	53,184111	4,847167	Yes	3	14-1-2016	9.7	10	Negative	Yes	Negative	NE	NAD
11	DE	13–1	54,085179	8,588861	No	4	16-1-2016	10.7	12	Negative	Yes	Negative	NAD	U
12	NL	14–1	52,994689	4,725887	No	4	External samples	11.5	NE	NE	NE	NE	NE	NE
13	UK	22–1	52,947346	0,488690	Yes	2	External samples	13.8	NE	Negative	NE	NE	NE	NE
14	UK	24–1	53,094011	0,337298	No	3 to 4	25-1-2016	14.6	NE	Negative	NE	Negative	NE	U
15	UK	24–1	53,094011	0,337298	No	3 to 4	External samples	14.7	NE	Negative	No	NE	NE	NE
16	UK	24–1	53,139982	0,349633	No	3 to 4	25-1-2016	13.5	NE	Negative	Yes	Negative	NE	U
17	UK	25–1	53,048060	0,263223	No	NE	NE	NA	NE	Negative	NE	Negative	NE	NE
18	DE	31–1	53,942594	8,900214	No	4	7-2-2016	10.8	12	Negative	Yes	Negative	NAD	U
19	DE	31–1	53,942594	8,900214	Yes	2	External samples	11.7	11	Negative	NE	NE	NAD	NE
20	DE	31–1	53,942594	8,900214	No	4	6-2-2016	11.2	10	Negative	Yes	Negative	NAD	U
21	DE	31–1	53,942594	8,900214	No	4	7-2-2016	11.0	12	Negative	Yes	Negative	NAD	U
22	DE	31–1	53,942594	8,900214	No	4	4-2-2016	10.2	10	Negative	Yes	Negative	NAD	U
23	DE	31–1	53,942594	8,900214	No	4	4-2-2016	11.3	15	Negative	Yes	Negative	NAD	U
24	DE	31–1	53,942594	8,900214	No	4	5-2-2016	11.4	11	Negative	Yes	Negative	NAD	U
25	DE	31–1	53,942594	8,900214	No	4	7-2-2016	10.5	12	Negative	Yes	Negative	NAD	U
26	FR	2–2	50,986444	1,959278	No	3 to 4	3-2-2016	13.85	NE	Negative	Yes	Negative	NE	U
27	DE	3–2	54,168224	8,733862	No	4	6-2-2016	12.0	11	Negative	Yes	Negative	NAD	U
28	DE	3–2	54,133607	8,654462	No	4	6-2-2016	11.4	15	Negative	Yes	Negative	MAD	U
29	UK	4–2	52,959184	0,502995	Yes	1	5-2-2016	13.6	N/E	Negative	Yes	Negative	NE	NAD
30	DK	25–2	55,562139	8,072900	No	NE	NE	NE	NE	NE	NE	NE	NE	NE

### Biological data

All whales were immature males (spermatogenesis not present or not extensive), with a straight-line body length ranging from 9.6 to 14.7 m and an average of 11.7 m. Age was determined in 20 cases, and ranged between 10 and 16 years (Table 1).

### Disease and pathogen investigation

#### Morphology and histopathology

From macroscopic evaluation, all necropsied animals were judged to be in a fair to good nutritional condition. In 9/22 cases marine debris was found in the gastro-intestinal tract [49]. No evidence of fractured or recently healed tympanic-periotic bone complex was detected in the thirteen animals investigated (ID # 1, 4, 5, 11, 18, 20–25, 27, 28 [46]). The state of preservation of most cases did not allow gas- and fat embolism analysis, but no visual evidence was found in the freshest cases (ID # 6–9, 29).

Histopathological findings included focal, severe dermatitis with epithelial degeneration in five sperm whales (ID # 6–10); congested dermal papillae in six sperm whales (ID # 11, 19, 21, 24, 25 and 28); and cutaneous haemorrhage with oedema in one (ID # 26). Five animals showed rake mark lesions on their tail flukes (ID # 4, 7, 18, 21, 23), one fresh enough to still be haemorrhagic upon discovery (ID # 23) while two others (ID # 18 and 21) presented bilateral and alternating lacerations, with inter-distance that resemble killer whale (*Orcinus orca*) interdental distances [62]. This suggests possible recent interactions between these species. One sperm whale had hyaline myofiber degeneration (Zenker’s degeneration, rhabdomyolysis) (ID # 4) and extensive muscle damage and degeneration was apparent in an additional four whales (ID # 6, 7, 9 and 26). Histologic assessment of the kidneys revealed intratubular protein leakage and intravascular microthrombi in one sperm whale (ID # 6). Two animals demonstrated congestion and intensive centrilobular necrosis of the liver (ID # 6 and 9). Severe pulmonary oedema; intra-alveolar haemorrhages or lung congestion was confirmed in four cases (ID # 6, 9, 24 and 26). Two whales showed lymphoplasmacytic inflammation with partly eosinophilic inflammation (ID # 4 and 21), one animal had an acutely haemorrhagic tongue (ID # 10) and finally another whale showed focal vacuolar degeneration of the palatum durum epithelium (ID # 9). All changes were either associated with the stranding process or unlikely to have been severe enough to cause stranding (S1 Table).

#### Parasitology

All necropsied sperm whales had infections with single or multiple parasite species, but all infections were considered of low intensity (S2 Table). Most frequently found were infections with larval stages of *Phyllobothrium delphini* in the blubber of 21 sperm whales (ID # 1, 2, 4, 5, 7, 8, 11, 13–16, 18, 20–25, 27–29); infections with anisakid nematodes belonging to the genus *Anisakis simplex sensu stricto* in six sperm whales (ID # 6, 8, 9, 11, 16 and 29); infections with acanthocephalans, morphologically and molecularly identified as *Bolbosoma capitatum*, in nine sperm whales (ID # 4–10, 18 and 25); infections with *Corynosoma curilensis* of two sperm whales (ID # 5 and 25); infections with *Chondracanthus lophii* of two sperm whales (ID # 6 and 7) and the detection of one *Pennella balaenopterae* associated with a severe, pyogranulomatous, deep dermatitis around the anchoring location in one case (ID # 28). No significant gross or histological changes of the organs with parasitic infections were detected.

#### Virology

Virological examination resulted in the discovery of a novel cetacean alphaherpesvirus from blowhole swabs of three sperm whales (ID # 18, 20 and 24). No macroscopic or histologic lesions were identified. Four other cases (ID # 6–9) investigated for the presence of Influenza A virus, morbillivirus and herpesvirus did not result in further viral detections (S2 Table), however, no blowhole swabs of these cases were available.

#### Microbiology

Microbiological data were considered as unspecific microbiota. Potentially pathogenic bacteria cultured were *Clostridium perfringens*, *Escherichia coli*, *Klebsiella pneumonia* and *Vibrio* spp., but no associated inflammatory lesions were found histologically. No potential zoonotic organisms such as *Brucella* spp. and *Erysipelothrix rhusiopathiae* were isolated.

### Diet

Previous consumption of squid was evident from large numbers of squid beaks in the gastrointestinal tract, the majority (97%) belonging to the Boreoatlantic armhook squid (*Gonatus fabricii*). The results of the diet studies reveal that the 13 sperm whales stranded in Germany (ID # 2, 4, 5, 11, 18–25, 27–28) contained 55,150 beaks, representing a cumulative prey biomass of around 12,000 kg; five sperm whales stranded in the Netherlands (ID # 6–10) contained over 11,000 lower beaks, resembling a prey biomass of around 2100 kg; and the one sperm whale that stranded in France contained nearly 33,000 lower beaks, resembling a prey biomass of around 5700 kg. Although there were a high number of squid beaks present in the gastrointestinal tracts of some individuals, they represented non-recent prey consumption, as the majority of the contents mainly consisted of dry squid beaks whilst the prey’s flesh was already digested.

### Environmental data

During the period of the strandings, from the end of 2015 through to the beginning of 2016, SST in the northern North Sea was only slightly higher than the average for that time of the year (Table 2, S2 Fig). The ocean currents were predominantly directing from west to east (Table 2), and this was therefore unlikely related to the sperm whale strandings.

**Table 2:** Sea surface temperature (SST) anomaly based on the daily average between 1981 and 2011 in °C and direction in degree angle and speed in m/s of ocean current on the position 61.28° N, 1.85° E retrieved from [https://earth.nullschool.net/] on the 22.06.2017; based on data from Ocean Surface Current Analyses Real-time (OSCAR) [http://www.esr.org/oscar_index.html] for ocean currents and for SST on data from NOAA (Marine Modelling and Analysis Branch of the Environmental Modelling Center within the National Centers for Environmental Prediction of the National Weather Service) [http://polar.ncep.noaa.gov/].

Date	Sea surface temperature anomaly	Origin of direction of ocean current and speed
30-11-2015	+0.3°C	240° at 0.03 m/s
05-12-2015	+0.5°C	280° at 0.14 m/s
10-12-2015	+0.4°C	285° at 0.06 m/s
15-12-2015	+0.2°C	290° at 0.16 m/s
20-12-2015	+0.8°C	285° at 0.11 m/s
25-12-2015	+0.3°C	260° at 0.15 m/s
30-12-2015	+0.0°C	280° at 0.18 m/s
05-01-2016	+0.5°C	280° at 0.18 m/s

One earthquake with a magnitude of 4.5 on Richter scale occurred on 18-12-2015 at 18:29:29.6 UTC, 284 km W of Lisbon, Portugal (39.58N;12.22W)[53]. Given its location in the southern North Atlantic Ocean, this was also unlikely to be related to the sperm whale strandings.

### Genetics and contamination profiles

Levels of a range of contaminants were determined, but were not considered to be of significance in terms of causality of the mortality event (details in [60]). Data on contaminant profiles together with genetic data gave evidence for at least two cohorts among the stranded sperm whales with different origins; one from the Canary Islands and one from the northern part of the Atlantic [59,60]. The genetic diversity was comparable to the genetic diversity in sperm whales from the entire Atlantic Ocean and males did not comprise maternally related individuals within this stranding event, but assemblages of individuals from different geographic regions [59].

### Historic data

**Fig 2:**
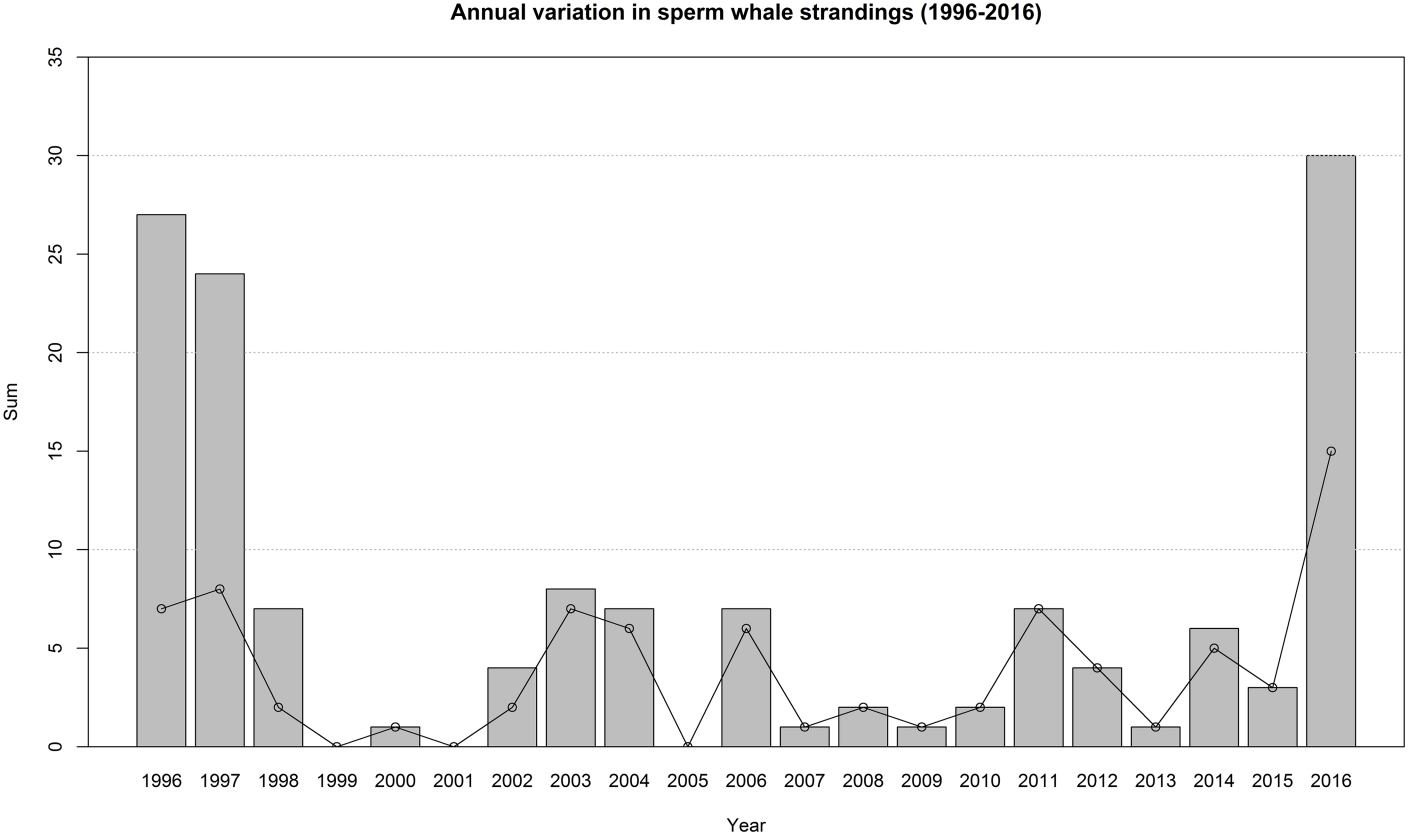
Annual variation in sperm whale strandings in the North Sea. Grey bars indicate the total number of stranded sperm whales per year; points and connecting lines show the total number of individual stranding events per year.

A total of 80 sperm whale stranding events were reported in the southern North Sea between 1996 and 2016, comprising 142 individual whales ((S3 Table) excluding Northern Scotland, Orkney and Shetland). Sixteen sperm whales stranded in a single mass stranding event on the Danish Wadden Sea island of Rømø on 27^th^ March 1996. On 4^th^ December 1997 another mass mortality event occurred on the island of Rømø, involving 13 sperm whales. That same year 11 additional sperm whales stranded in eastern Scotland, England, The Netherlands and Germany, making 1996 and 1997 the years with high stranding numbers, followed by 18 years of relatively low stranding numbers, until the mortality event of 2016 (Fig 2).

## Discussion

Here we describe the largest recorded sperm whale mortality event in the southern North Sea, where a total of 27/30 of the stranded sperm whales were necropsied and/or sampled. The cohesive examination of the mortality event across the North Sea region represents the most extensive investigation of a sperm whale mortality event ever undertaken on a global basis. Through the pathological examination, and subsequent ancillary tests, we are able to eliminate poor health status or an identifiable traumatic or infectious disease as the primary driver of this mortality event. Infectious agents, including the novel identified species of herpesvirus, had not impacted the health status of the animals. Marine litter was found in 9/22 sperm whales, but had not caused a functional obstruction and was thus not deemed to be a factor in the death of these animals [45]. Elimination of a deteriorated health status and disease as a common cause of these strandings suggest that other factors, including previously suggested environmental and anthropogenic causes of mass strandings, were plausibly more influential.

### Anthropogenic factors

Previous mass strandings of beaked whales have been linked to high intensity sources of marine noise, including naval sonar, which probably led to behavioural responses resulting in gas- and fat embolism [17,19]. Cases diagnosed with gas embolism have occasionally been reported in stranded cetaceans from the North Sea region [19,63]. No evidence of significant pathology indicative of gas embolism was found in the fresh sperm whales examined, however it was impossible to assess this for the majority of the stranded sperm whales as they were in an advanced state of decomposition. Additionally the structural analysis of the tympanic bullae of several cases [46] demonstrated no severe damage. Assessment of the tympanic bullae for any evidence of damage to or loss of the hair cells in the cochlea could not be carried out due to the decomposed condition of the animals.

Anthropogenic acoustic data (e.g. high-intensity naval sonar/activities) and other sources of noise pollution could not be readily assessed in this mortality event. It is impossible to relate acoustic activity to the mortality event as in the North Sea and North East Atlantic an effective register of anthropogenic impulsive noise inputs was lacking. Besides it remains unknown when exactly the sperm whales entered the North Sea or how long they may have been resident there.

Persistent organic pollutants (POPs), particularly polychlorinated biphenyls (PCBs), negatively affect a variety of cetacean species (e.g. [20]). Levels of a range of potential contaminants were determined in samples collected from sperm whales in this study, but were not deemed to be of significance in terms of direct causality of the stranding event [60].

### Environmental factors

Environmental factors that have been proposed to cause mass strandings of cetaceans include harmful algal blooms (HABs, e.g. [64]) and earthquakes (e.g. [65]). While there has not been an assessment of exposure to biotoxins from HABs in the sperm whales from this mortality event, this is an unlikely cause of the strandings. The strandings occurred in the middle of the winter, with HABs commonly occurring in the warm-water seasons. Only one earthquake with a magnitude of 4.5 on Richter scale was reported in the southern part of the northern Atlantic Ocean in December 2015. A correlation with the mortality event cannot fully be ruled out, however this seems unlikely as the location of the earthquake was approximately 2500–3000 km away from the northern entrance to the North Sea and occurred three weeks prior to the first stranding event. In addition, and according to the authors’ knowledge, no other increases in sperm whale strandings, or other species, along the east Atlantic coastline have been reported.

Pierce et al. [6] previously reported a potential relationship between increases in SST and sperm whale stranding rates. This explains 8–9% of the variation in sperm whales strandings in the North Sea. In the period prior to the 2016 strandings, SST of the North Sea was slightly higher than on average for that time of the year. When comparing the SST of years with high and low frequencies of strandings it becomes evident, however, that no obvious trend exists. For the period in 1996, during which a large number of sperm whale strandings was recorded, SST was below the long-term average, whereas in the period before the 2016 strandings, SST was above the average. In years without strandings, we also found no obvious pattern. The potential relationship between climatic variation, including SST, and sperm whale strandings therefore requires further investigation using longer term datasets, to be able to recognize any trends [6]. This should also be done in light of climate change, which may alter prey availability and distribution [66].

Changes in solar activity have recently been proposed as an explanatory factor for sperm whale stranding events, including those in 2016 [67]. Solar storms can alter the earth’s magnetic field and impact the ability of migratory species to orientate accurately by means of magneto-reception. Vanselow and Ricklefs [22] showed that between 1712 and 2003 up to 90% of sperm whale strandings occurred during solar cycles of length less than 11 years. We are currently experiencing the end of the 24th solar cycle which began in December 2008 [68]. This solar cycle represents the weakest cycle of the last 115 years [69], yet it coincides with the largest recorded stranding event of sperm whales in the North Sea. Vanselow et al. [67] therefore recently moved from investigating entire solar cycles to investigating the relationship between short term solar activity and their potential effects on the earth’s magnetic field. The authors concluded that a single solar storm can shift the magnetic field by up to 460 km for a period of up to a day and may cause sperm whales to deviate from their usual migratory routes into the North Sea and propose this as a possible explanation for the 2016 stranding event. On the other hand preliminary analytical results conducted by NASA in cooperation with the International Fund for Animal Welfare (IFAW) were unable to find any clear causal connection between geomagnetic activity and mass stranding in the vicinity of Cape Cod. However they could not exclude solar weather (or geomagnetic activity) as one of several contributing factors to these events (Personal Communication Katie Moore IFAW). The stranding records for sperm whales in the North Sea represent the longest time series available for any cetacean [6] and the addition of two decades of further data to the historic database initiated by Smeenk [5] as presented here, will help facilitate further in-depth analyses regarding this topic.

### Prey

Sperm whales in the Northeast Atlantic mainly feed on *Gonatus fabricii* [70]. Similar to previous studies (e.g. [48,71]) all sperm whales had squid beaks in their stomachs. Almost all beaks were identified as *G*. *fabricii*, which is distributed throughout the deep and cold waters of Arctic and subarctic regions [70,72]. The southern distribution boundary is around 61° N [73,74]. The main spawning time of *G*. *fabricii* depends on the geographical location and occurs between December and February for the area of the eastern Norwegian Sea [74]. Prior to spawning at depth female *G*. *fabricii* lose their ability to actively swim as an effect of degeneration of their muscle tissue and subsequently drift in large groups [70,75,76]. This could be used by cetaceans, such as the sperm whale, as an easy-to-access resource [70]. The diet study revealed relatively high (compared to other prey) reconstructed biomass of *G*. *fabricii* in the sperm whales’ gastrointestinal tracts, but concluded that the majority of the contents found consisted of dry squid beaks which were not recently ingested. The distance between the southern distribution boundary of *G*. *fabricii* and the stranding locations of the sperm whales in the southern North Sea is ~1300 km. Existing records for swimming speeds of sperm whales vary, from 2.9 km/h as a minimum [77] to 5.4 km/h as a maximum [78]. A sperm whales’ travel time over a distance of ~1300 km would therefore take minimally 10 days (at a speed of 5.4 km/h) and maximally 19 days (at a speed of 2.9 km/h), making it highly likely that these animals had not fed substantially recently, or at least not within the minimal period of 10–19 days prior to their stranding. This also suggests that squid beaks may be retained in sperm whale stomachs for a longer period than previously estimated by Clark [51] which indicated squid beaks retention times varying from 2.1 to 2.6 days in female sperm whales, to 1.2 to 1.6 days in males.

### The North Sea region

Mass mortalities of sperm whales have occurred on the beaches of the (southern) North Sea for centuries. These stranding locations present one common feature: gradually sloping sandy coastlines. The characteristics of the stranding sites are very similar to the characteristics of other stranding sites where mass mortalities involving sperm whales occurred, like along the Italian side of the Adriatic Sea [26], and in New Zealand and South Australia [26,79]. It has been theorized that strandings of deep diving pelagic cetacean species are centred around such ‘acoustical dead zones’ as a result of distorted echolocation signals due to geometric effects [79].

**Fig 3:**
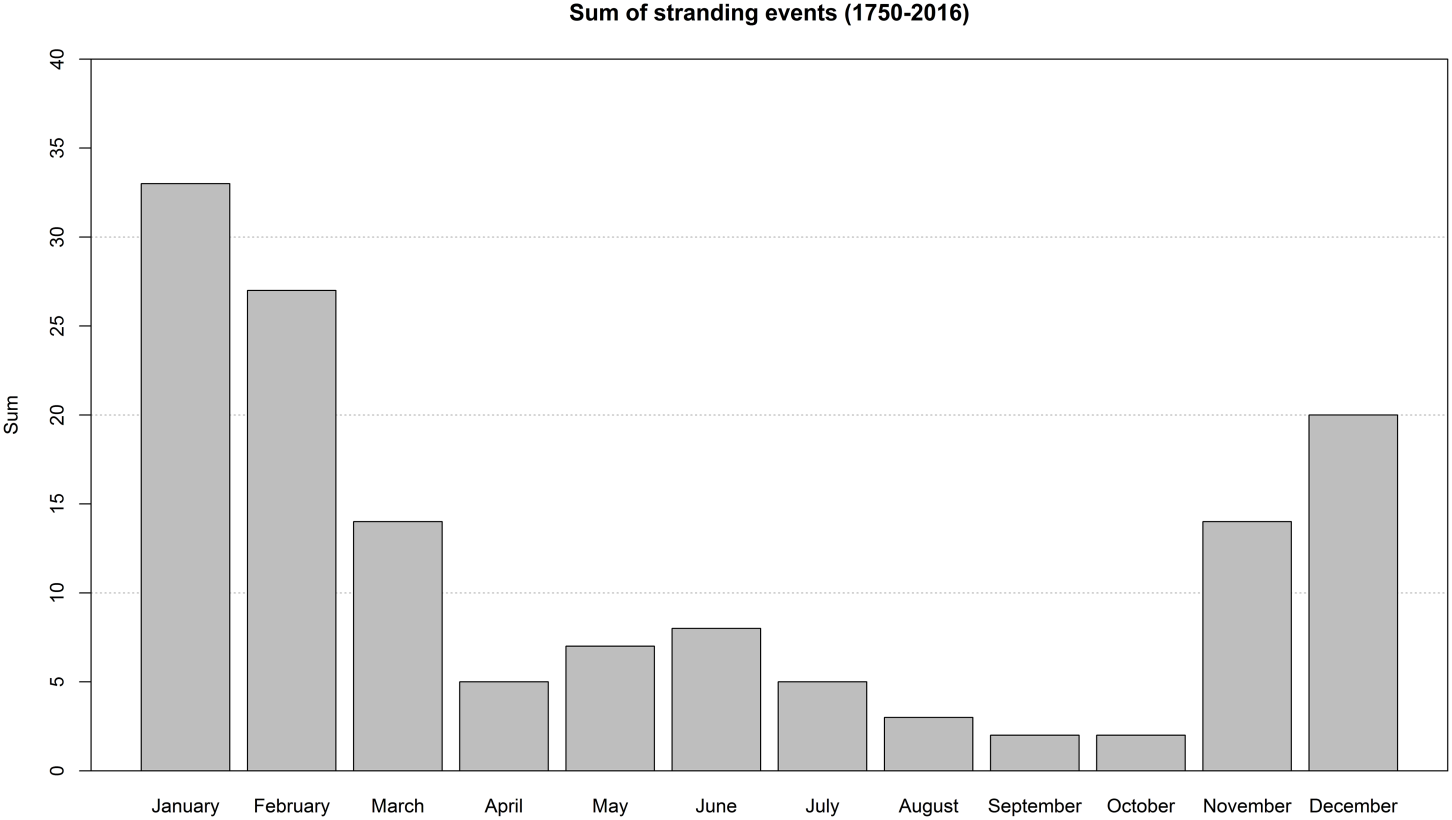
Seasonal pattern of sperm whale stranding events in the southern North Sea (1750–2016). Numbers prior to 1996 derived from Smeenk [5].

It is likely that stranding events in the North Sea have largely been well documented over the past 400 years, providing reasonably accurate stranding records over time. Several periods with relatively large numbers of strandings have been previously identified in 1560–1995 [5,80]. During the 20^th^ century sperm whale mortality events in the North Sea always involved males, primarily immature individuals, which stranded predominantly between November and March ([5,6,21,81], and the CSIP and Naturalis databases; Fig 3). The 2016 mortality event fits into this pattern, with all animals being immature males that stranded in winter. Some historical mortality events stretched over entire winter or spring periods, whereas the event we describe here occurred over a period of only six weeks.

Smeenk [5] and Evans [82] described an increase in sperm whale stranding events in the North Sea since the 1970s, and reported through into the 1990s. There has been a general increase in sperm whale strandings around the southern North Sea region over the last ~30 years (S3 Table), with several years of extremely high numbers (1996, 1997 and 2016) and multiple years with low or no strandings (e.g. 1999–2001, 2007–2009). There is a possible relationship between the frequency of strandings and the number of males present at northern latitudes, which might be evidence of some degree of population recovery resulting in an increased number of migrating males [82]. However without accurate population estimates, drawing such assumptions should be treated with caution [5]. Altogether, this emphasises the need for multidisciplinary, cross-boundary, and even cross-region investigations into such mass mortality events and hotspots, especially taking into account changed climatic conditions and increased anthropogenic activities in sperm whale habitats.

## Conclusion

In order to elucidate potential reasons for the largest sperm whale stranding event that has ever been recorded in the North Sea we conducted a systematic pathological examination of the majority of the stranded whales, leading to one of the most extensively investigated sperm whale mortality events that has ever taken place. This allowed for a thorough assessment not just into the cause of death and stranding, but also the health of a group of animals almost impossible to study by other means. We are able to eliminate poor health status, any identifiable traumatic event or significant infectious disease process as the primary cause of the 2016 sperm whale strandings. Although all investigated animals had high reconstructed squid biomass in their stomachs we conclude that they did not ingest these recently and not within the North Sea. Whilst the ultimate reason why these animals entered the North Sea remains unknown, and it is impossible to rule out whether these stranding events are just a result of ‘bad luck’ after a navigational fault of (some of) the animals, this was an unprecedented event and enabled a number of potential hypotheses, both natural and man-made, to be excluded. We conclude that no single causal factor for this series of strandings can be identified and it is certainly plausible that a combination of different and coincident factors may have led to this large-scale sperm whale mortality event. A summary of all investigations and their outcomes can be found in S4 Table. It is only through multidisciplinary, collaborative approaches that potentially multifactorial large-scale stranding events can be effectively investigated, and significant causes appointed or excluded.

## Supporting information

S1 Table Overview of pathological findings. NE = Not Examined. NAD = No Abnormalities Detected. NF = Not Found.
(XLSX)

S2 Table Overview of ancillary diagnostic tests. Virological, bacteriological and parasitology results of cases investigated. NE = Not Examined. NF = Not Found.
(XLSX)

S3 Table Sperm whale strandings North Sea region. Overview of all stranded sperm whales along the North Sea coastline (n = 80) from 1997–2016.
(XLSX)

S4 Table Potential drivers of the stranding events, with conclusions on the likelihood of causality for each factor. Colours reflect whether factors could be excluded (green), were unlikely (light green), or remain uncertain/could not be excluded (orange). In blue the most likely explanation for the stranding events, although the bathymetry of the region does not explain why the animals entered the North Sea.
(DOCX)

S1 Fig Bathymetry of North Sea region and route of sperm whales. The colour palette represents the total depth of the area, revealing depths of ~500 m in the Faeroe-Shetland channel, depths of approximately 200–400 m in the Norwegian trench, which decrease significantly in the central North Sea to 40 m. Here, sandbanks and coastal areas of 5 m depth are common. The green arrows indicate the route that sperm whales take during their southern migration through the Faeroe-Shetland channel. The red arrow indicates the most likely route sperm whales mistakenly take, by which they enter the North Sea region.
(TIF)

S2 Fig Sea surface temperature (SST) and sperm whale strandings. Averaged SST (green line) for the winters with high sperm whales stranding numbers in the North Sea region: 29th of November of the years 1994/95, 1995/96, 1996/97, 1997/98, 2013/14, 2014/15, 2015/16, 2016/17. The dotted line represents the respective confidence interval and the red coloured areas represent the difference of the measured temperature to the long-term. The area between the upper border of the red coloured areas and the green line represents the respective long-term SST average for this period. The blue triangles indicate the exact stranding events of sperm whales for each year in which they occurred.
(TIF)
