# Supplementary material for: Beached bachelors: An extensive study on the largest recorded sperm whale Physeter macrocephalus mortality event in the North Sea
Source: PLoS One. 2018 Aug 7;13(8):e0201221. doi: 10.1371/journal.pone.0201221 (PMC6080757; doi:10.1371/journal.pone.0201221)
Supplement: S4 Table — Colours reflect whether factors could be excluded (green), were unlikely (light green), or remain uncertain/could not be excluded (orange). In blue the most likely explanation for the stranding events, although the bathymetry of the region does not explain why the animals entered the North Sea. (DOCX) [file pone.0201221.s004.docx]

| **Potential drivers of the stranding events** | | **Main conclusion** | **Causality?** |
| --- | --- | --- | --- |
| **Anthropogenic factors** | Bycatch/entanglement | No evidence of acute or chronic net entanglement was detected in the individuals investigated. | Excluded |
|  | Marine debris | Marine debris was detected in 9/22 investigated individuals, but without associated pathology, therefore, very unlikely to be related to the death of the individuals assessed. | Excluded |
|  | Chemical pollution | A range of contaminants were determined, but no evidence was found that these had an influence on the health status of the individuals assessed. | Unlikely |
|  | Marine noise | No noise register was available; unable to assess gas- and fat embolism or histological auditory trauma due to the state of decomposition of most individuals. | Remains uncertain |
| **Environmental factors** | Epizootic or other infectious disease | All pathological changes in the assessed individuals were either associated with the stranding process or unlikely to have been severe enough to cause stranding. | Excluded |
|  | Earthquakes | One earthquake (magnitude of 4.5) in southern part of the N Atlantic Ocean was reported 3 weeks prior to, and 2500-3000 km away from the first event. | Unlikely |
|  | Harmful algal blooms | The stranding events occurred in winter when HABSs are unlikely to occur. | Unlikely |
|  | Sea surface temperature (SST) | In the period prior to the strandings, SST was slightly higher than on average for that time of the year, however, no trend was discovered when assessing long term sperm whale stranding records in relation to SST anomalies. Further research is however needed. | Unlikely |
|  | Solar storms | Conflicting conclusions are given by others (e.g. Vanselow et al. [62] and IFAW, personal communication) therefore at this stage no further conclusions can be drawn here. | Remains uncertain |
|  | Feeding/abnormal prey distribution | No consistent evidence of ingestion of prey at least 10 days prior to stranding. No data on (changes in) prey distribution. Therefore at this stage no further conclusions can be drawn here. | Remains uncertain |
| **Bathymetry** | Geometric effects | North Sea coastlines predominately consist of gradually sloping sandy beaches and could be considered ‘acoustic dead zones’ for sperm whales and other deep diving species. | Very likely related to the stranding events, but does not explain why the sperm whales initially entered the North Sea. |

**S4 Table. Potential drivers of the stranding events,** with conclusions on the likelihood of causality for each factor. Colours reflect whether factors could be excluded (green), were unlikely (light green), or remain uncertain/could not be excluded (orange). In blue the most likely explanation for the stranding events, although the bathymetry of the region does not explain why the animals entered the North Sea.
