# Supplementary figures and images for: Beached bachelors: An extensive study on the largest recorded sperm whale Physeter macrocephalus mortality event in the North Sea
Source: PLoS One. 2018 Aug 7;13(8):e0201221. doi: 10.1371/journal.pone.0201221 (PMC6080757; doi:10.1371/journal.pone.0201221)

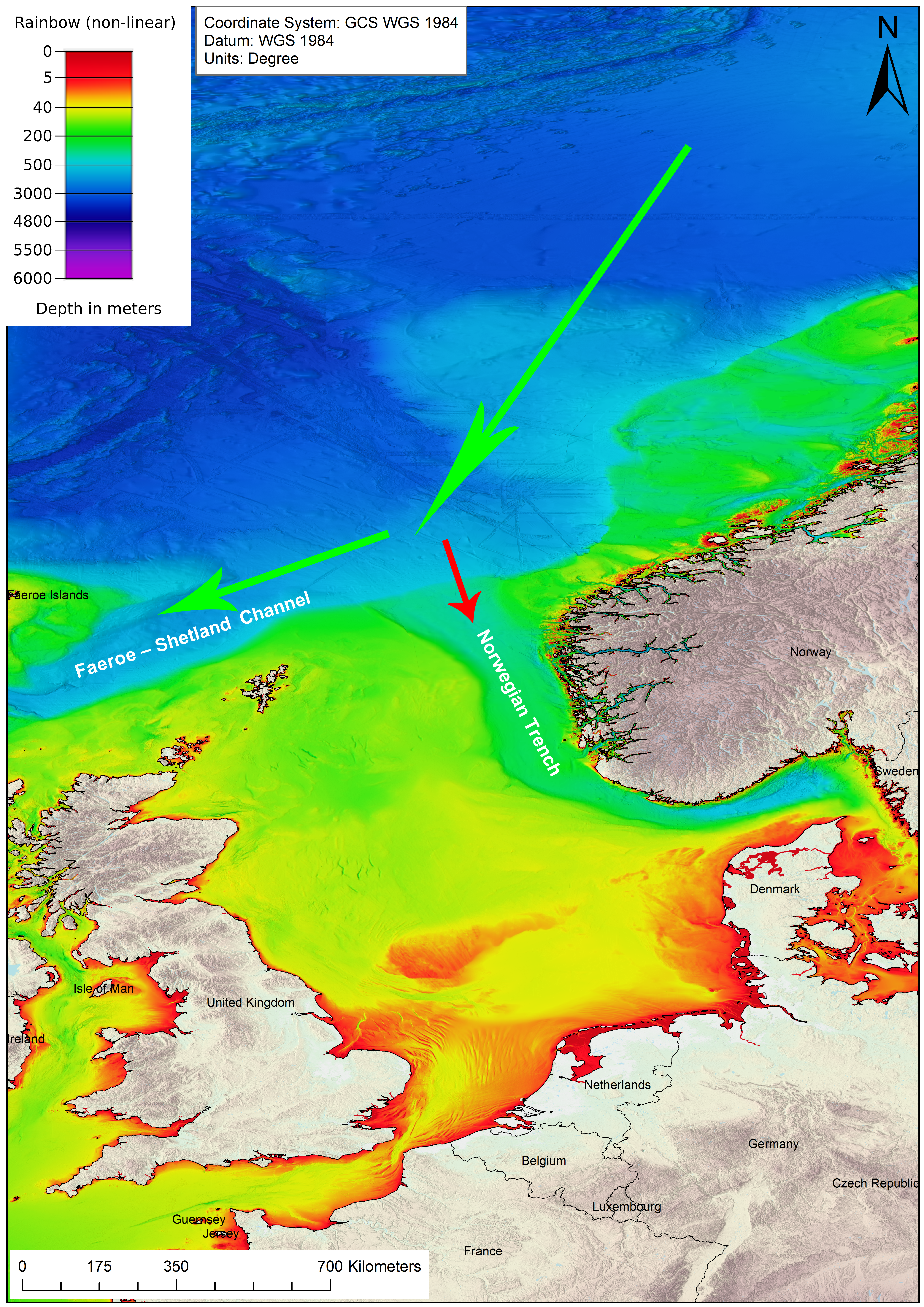

Supplement: S1 Fig — The colour palette represents the total depth of the area, revealing depths of ~500 m in the Faeroe-Shetland channel, depths of approximately 200–400 m in the Norwegian trench, which decrease significantly in the central North Sea to 40 m. Here, sandbanks and coastal areas of 5 m depth are common. The green arrows indicate the route that sperm whales take during their southern migration through the Faeroe-Shetland channel. The red arrow indicates the most likely route sperm whales mistakenly take, by which they enter the North Sea region. (TIF) [file pone.0201221.s005.tif]

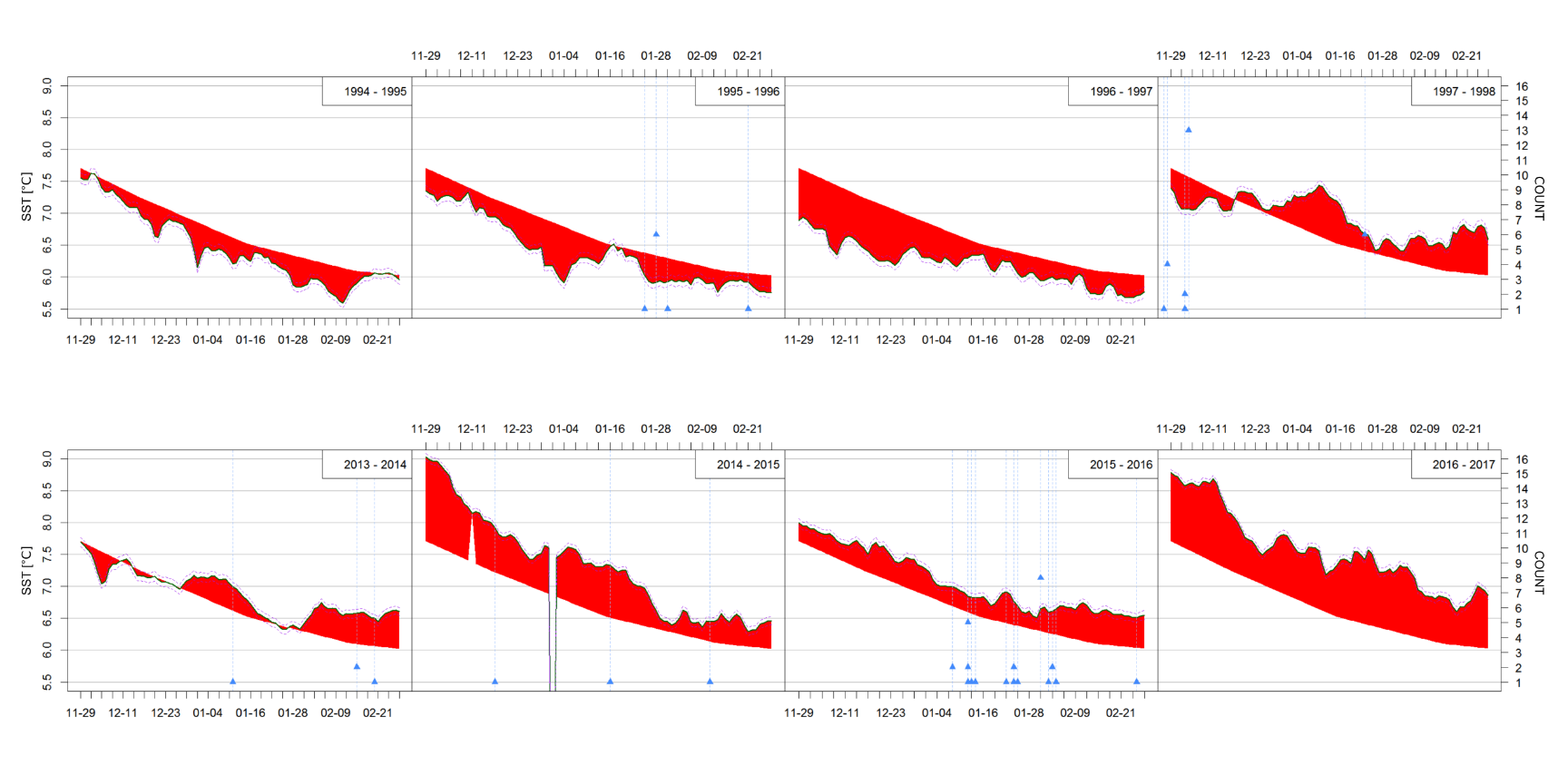

Supplement: S2 Fig — Averaged SST (green line) for the winters with high sperm whales stranding numbers in the North Sea region: 29th of November of the years 1994/95, 1995/96, 1996/97, 1997/98, 2013/14, 2014/15, 2015/16, 2016/17. The dotted line represents the respective confidence interval and the red coloured areas represent the difference of the measured temperature to the long-term. The area between the upper border of the red coloured areas and the green line represents the respective long-term SST average for this period. The blue triangles indicate the exact stranding events of sperm whales for each year in which they occurred. (TIF) [file pone.0201221.s006.tif]
